# Case Report of Kikuchi-Fujimoto Disease from Sub-Saharan Africa: An Important Mimic of Tuberculous Lymphadenitis

**DOI:** 10.1155/2020/4385286

**Published:** 2020-01-05

**Authors:** Karishma Sharma, Fredrick Otieno, Reena Shah

**Affiliations:** Department of Medicine, Aga Khan University Hospital, Nairobi, Kenya

## Abstract

Kikuchi-Fujimoto disease (KFD) is a rare form of painful lymphadenopathy, usually cervical, which is more common in Southeast Asia and rarely reported from Africa. Symptoms are usually nonspecific (fever, night sweats, etc.), and can mimic more common diseases such as tuberculosis (TB) in endemic areas. We report a case of a 29-year-old black African woman who was admitted with headache, neck pain, fever, and lymphadenopathy. She was found to have aseptic meningitis, eventually attributed to TB based on cervical node biopsy, although further histology suggested KFD. Blood tests for systemic lupus erythematosus (SLE) were negative; she had already been commenced on anti-TB treatment and had responded well and so was continued with this therapy. She was also later diagnosed with Hashimoto's thyroiditis 3 months after her diagnosis of KFD. Five months after stopping TB treatment, she was readmitted with the same symptoms and associated painless lymphadenopathy. Repeat biopsy was morphologically similar to that of 2017, and repeat evaluation confirmed SLE. She has since been managed by a rheumatologist and continues to do well.

## 1. Introduction

Kikuchi-Fujimoto disease (KFD) is a rare form of painful lymphadenopathy which is more common in Southeast Asia. KFD is not very well described in Africa, with very few reports described in the literature. The lymphadenopathy is usually cervical with associated nonspecific symptoms of fever and night sweats, which makes the diagnosis more challenging as the differential diagnosis is wide. We describe a young female, who presented with aseptic meningitis initially thought to be due to tuberculous meningitis and later confirmed to have KFD on lymph node biopsy, and subsequently diagnosed with Hashimoto's thyroiditis and systemic lupus erythematosus (SLE) 3 and 18 months after initial presentation.

## 2. Case Presentation

A 29-year-old black African woman presented to the emergency department with headache and fever for 5 days. Brain imaging was normal, and lumbar puncture for cerebrospinal fluid (CSF) analysis showed a raised white cell count (WBC) of 31/mm^3^ with 95% lymphocytosis, elevated CSF protein, and borderline high CSF adenine deaminase levels of 18.1 U/L. CSF Ziehl–Neelsen stain, GeneXpert, and culture for *Mycobacterium tuberculosis* (TB) were negative. Blood tests are in accordance with Table 1. Her headache and fever resolved with oral paracetamol, and she was discharged with a diagnosis of aseptic meningitis.

**Figure 1:**
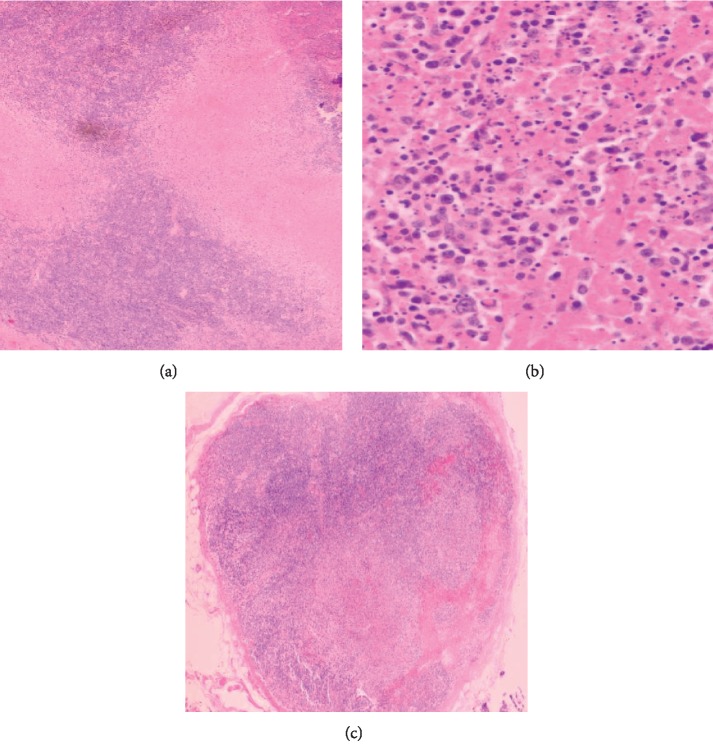
(a) Lymph node biopsy 2017 (low-power view): partial effacement of architecture with large area of necrosis with no acute inflammatory cell infiltrate in keeping with Kikuchi's disease. (b) Lymph node biopsy 2017 (high-power view): numerous apoptotic bodies with histiocytes. (c) Lymph node biopsy 2019: numerous foci of necrosis with apoptotic bodies with scattered histiocytes. Absence of acute inflammatory infiltrate and hematoxylin bodies in keeping with Kikuchi's disease.

However, she was readmitted with worsening neck pain and headache 2 weeks later and was found to have significantly enlarged cervical lymph nodes. Further evaluation with an MRI scan of the neck showed features in keeping with TB lymphadenitis. A repeat of laboratory workup (refer to Table 1) remained negative. She also had a positive EBV IgG but with a negative serum IgM. A cervical lymph node was biopsied. Histopathological examination revealed necrotizing lymphadenitis with no granulomas (Figures 1(a) and 1(b)). Since the histologic appearance was indistinguishable from that of TB lymphadenitis, the patient was commenced on TB treatment (ATT) and was reviewed in a clinic 3 weeks later and found to have improved significantly.

The slides were sent for a second opinion overseas (USA), where the cervical biopsy was noted to show marked architectural effacement with large areas of necrosis containing abundant apoptotic debris. The adjacent viable areas contained numerous histiocytes, small lymphocytes, occasional plasma cells, and immunoblasts (Figures 1(a) and 1(b)). Hematoxylin bodies were not identified. Immunohistochemistry performed on the slides was CD20/PAX5 (B-cell marker)-positive, CD68 (histiocyte marker)-positive, and CD123 (plasmacytoid dendritic cells)-positive with a Ki of 67–60%.

These findings were in keeping with a diagnosis of histiocytic necrotizing lymphadenitis likely secondary to either KFD or SLE. However, given the patient was responding well to the TB therapy and she had screened negatively for SLE, she was continued on the treatment. Three months into her TB treatment, she was found to have an elevated thyroid stimulating hormone (TSH) of 47 pg/ml with low thyroxine and thyronine levels and positive antithyroid peroxidase and thyroglobulin antibodies in keeping with Hashimoto's thyroiditis. The patient completed 12 months of TB treatment and remained well and was discharged.

However, 5 months later, she presented to the infectious disease's clinic with a 1-month relapse of the same symptoms. She reported a history of travel to a malaria-endemic region 2 weeks prior and had taken a complete course of self-prescribed antimalarial medication with no resolution of symptoms. On examination, she had a temperature of 38°C but otherwise normal vital signs and large >1 cm bilateral nontender nonmatted upper cervical lymphadenopathy, but otherwise a normal physical examination with no meningism.

Investigations on this occasion are summarized in Table 1.

Interestingly, the repeat autoimmune screen revealed ANA positive with titers of 1 : 320 and a speckled pattern. Her ENA anti-SS-A/RO-52 was also positive. The remainder of her evaluation was unremarkable except for persistent leucopenia, anemia, and thrombocytopenia.

She was reviewed by the rheumatologist and diagnosed as a case of systemic lupus erythematosus after she met 4 components of the SLICC criteria which included thrombocytopenia, leucopenia, arthritis, and positive ANA. Her Anti-Ro antibody was also positive and even though not part of the SLICC criteria is still in keeping with the diagnosis of SLE. While in hospital, her fevers settled spontaneously with antipyretics. At the time of discharge, she was initiated on prednisolone, azathioprine, and hydroxychloroquine which she is currently on and is asymptomatic. She currently remains on follow-up in the Rheumatology clinic.

## 3. Discussion

Kikuchi-Fujimoto disease, also known as histiocytic necrotizing lymphadenitis, was first described in 1972 [1]. It is a rare self-limiting lymphadenopathy, more commonly described in females, those aged 20–40 years and are of Southeast Asia origin [2, 3]. Although less common within the African population, a few cases of the disease have been reported from Gabon, Senegal, Morocco, and Tunisia [4–6]. The disease affects both adults and children. The sex ratio is variable in the pediatric age group with males affected more than females, which is reversed in adults [7].

The exact pathophysiology of KFD is not known, but the two theories postulated are infectious and autoimmune etiology [8]. Xu et al. identified CMV,EBV, HHV-6, HHV-7, Parvovirus B19, and mycobacterial species from lymph node samples of 153 patients in China with KFD [9]. KFD has been described in association with SLE, Still's disease, Wegener's granulomatosis, Graves' disease, and Sjogren's disease [3]. Tanaka et al. found higher frequencies of the HLA DPA1*∗*01 and DPB1*∗*0202 allele in Japanese patients with KFD [10]. Familial cases of KFD have been reported in the literature [11].

The largest published series of 244 KFD cases has shown that fever (35%), fatigue (7%), and joint pain (7%) are the most frequent symptoms, and lymphadenopathy (100%), leucopenia (43%), high erythrocyte sedimentation rate (40%), anemia (23%), and erythematous rashes (10%) are the most common findings [12]. Our patient had high fever, lymphadenopathy, leukopenia, anemia, and high ESR. Cervical lymphadenopathy is the commonest site for lymphadenopathy and is usually painful [13].

The clinical picture of KFD is often nonspecific and can be in keeping with several diagnoses such as infectious mononucleosis, bacterial lymphadenitis (mainly tuberculosis which is highly endemic in Africa), malignant lymphoma, or metastatic cancer [12]. The nonspecific nature of presentation is associated with diagnostic delay and misdiagnosis of patients [14, 15], especially in TB endemic regions such as Kenya, where patients get unnecessary ATT treatment.

Baenas et al. noted three patterns of presentation of association between KFD and SLE which are before the onset of SLE (30%), simultaneous occurrence of both disorders (47%), and KFD after SLE (23%) [16]. Goldblatt et al. reported 4 cases of patients who developed SLE after 4–13 months of diagnosis with KFD [17]. Since SLE evolves over many years and patients may not meet the diagnostic criteria at onset or in the initial phases of the disease, it is likely that the initial diagnosis of KFD in this patient may have been an attenuated form of SLE [18]. However, it remains difficult to reach this conclusion since the diagnosis is dependent on standard diagnostic criteria which our patient did not meet until much later in the disease. Interestingly, she has not had any flares of her disease following the initiation of her treatment for SLE, and it remains unclear whether her initial response to the anti-TB medication was more due to the steroids used in the treatment of CNS tuberculosis. Due to the association of KFD with SLE, follow-up and screening of patients with KFD for later diagnosis of SLE are recommended [19].

CNS manifestations of KFD are rare, and so far, aseptic meningitis, encephalitis, and cerebellar ataxia have been reported in the literature [20, 21]. Aseptic meningitis is the commonest neurological presentation of Kikuchi's disease [22]. The CSF white cell count may range from 60 to 170 mm^3^ and may present as recurrent meningitis within intervals of 1 month–11 years [23]. The diagnosis of KFD as a cause of the aseptic meningitis is made following exclusion of other potential reversible causes.

Hashimoto's thyroiditis has also been associated with Kikuchi disease. In majority of the cases, KFD and Hashimoto's thyroiditis occurred concurrently; however, in three cases, the diagnosis of Hashimoto's thyroiditis preceded that of KFD [24]. Previous reported cases note that it is more common in females and may be positive for ANA, complement, and antiphospholipid antibodies. Most reported cases complete resolution within a year following supportive care in some patients and steroids in others [24]. We came across a case report of a 17-year-old female with concurrent SLE and Hashimoto's thyroiditis who responded well to steroids and hydroxychloroquine. [25] Similarly, Aqel et al. reported a case of KFD in Saudi Arabia with Hashimoto's thyroiditis and mixed connective tissue disease [26].

The diagnosis of Kikuchi-Fujimoto disease is made on biopsy. Histologically, lymph nodes usually have partially preserved architecture with follicular hyperplasia. Necrotic foci show abundant karyorrhectic nuclear debris and a large accumulation of histiocytes at the edge of necrosis. The histiocytes in KFD are positive for lysozyme, myeloperoxidase, CD68, CD163, and CD4. The lymphocytes in the lesions are mostly CD3-positive T cells demonstrating a predominance of CD8 compared with CD4, with very few CD20-positive B cells [3]. In contrast, lymphadenitis from TB classically has the presence of well/poorly defined granulomas with areas of caseous necrosis [27]. The proliferative stage of KFD may be confused with lymphoma due to presence of large atypical cells and immunoblasts of T-cell lineage origin [18].

There is no consensus regarding treatment for this disease. Patients with mild disease respond to supportive care with antipyretics and NSAIDs. In contrast, those with extranodal disease with CNS and lung involvement may respond to short courses of corticosteroids [28].

During the first admission, the patient was on dexamethasone as part of her TB meningitis treatment. This could explain the initial resolution of her symptoms. Irrespective of the form of intervention, the prognosis of the condition is reported as favorable. However, there have been reported cases of death especially in patients who develop DIC [29].

Song et al. reported a higher incidence rate of relapse in ANA-positive cases and identified fever, fatigue, extranodal involvement, and positive fluorescence antinuclear antibody as predictive factors for relapse [30].

To the best of our knowledge, very few cases of this disorder have been reported so far from Africa. The challenges with making this diagnosis we believe are multiple including lack of clinician familiarity with disorder, few case reports, nonspecific signs and symptoms, poor laboratory support, and presence of alternative diagnosis especially tuberculosis in our set up.

## Figures and Tables

**Table 1:** Laboratory results summarized from 2017 to 2019.

Variable	Reference range	Aug 2017	Sept 2017	Jan 2018	July 2018	Feb 2019
White blood cell	4–10 (*∗*10^9/L^)	2.51	3.19			2.06
Neutrophil count	2–7 (*∗*10^9/L^)	1.16	2.12			1.14
Lymphocyte count	1–3 (*∗*10^9/L^)	1.10	0.81			0.80
Hemoglobin	11.5–16.5 (g/dl)	10.2	11			11
Platelet count	150–450 (*∗*10^9/L^)	118	153			85
ESR (Erythrocyte sedimentation rate)	0–20 (mm/hr)		44			61
CRP (C-reactive protein)	0–5 (mg/l)	1.63	15.1			
AST (aspartate transaminase)	0–35 (U/L)	22	23.5			33.4
ALT (alanine transaminase)	0–35 (U/L)	17.7	13.9			15.6
Creatinine	58–98 (micromol/l)	65	59			60
HIV Elisa		Neg	Neg		Neg	
CSF:						
Cell count	0–5 (mm^3^)	31.25	295			<5
Neut	%	6	4			
Lymph	%	94	96			
Glucose	2.22–3.89 (mmol/l)	2.39	3.51			2.69
Protein	0.15–0.45 (g/l)	0.52	0.56			0.44
Tb Gene expert			Neg			Neg
CSF adenine deaminase	0–5 (U/L)		18.41			0.29
CSF culture		No growth	No growth			No growth
CSF viral PCR-herpes + enterovirus		Neg				Neg
EBV (Epstein-Barr virus)		IgG-positive IgM-neg				
Tropical fever screen- (i) Malaria antigen (ii) Leptospira (iii) Rickettsia (iv) Dengue (v) West Nile virus (vi) Chikungunya		Neg				Neg
Malaria parasite		Absent	Absent			
Blood culture		No growth				No growth
Urine culture		No growth				No growth
ANA (antinuclear antibody)			Neg			Positive 1 : 320 speckled
ENA						Positive for SS-A/RO-52
Complement 3/4						Normal
Anti-dsDNA			Neg			Neg
Antiphospholipid antibody						Neg
Direct Coombs test						Neg
Ferritin	13–150 (ng/ml)					Normal
Lipid profile						Normal
TSH (thyroid stimulating hormone)	0.27–4.2 (pg/ml)		1.68	49.77	25.24	4.23
T3	2–4.4 (pg/ml)		2.88	1.67	2.97	1.87
T4	0.97–1.58 (ng/ml)		0.87	0.32	0.84	0.86
Anti-TPO antibodies	<34 (IU/ml)				290	
Antithyroglobulin	<115 (IU/ml)				226	
TSH receptor antibodies	<1.22				0.468	
Lymph node biopsy			Refer to Figures 1(a) and 1(b)			Refer to Figure 1(c)
Tissue Tb culture			Neg			
Bone marrow aspirate						Normocellular

## References

[1] Lame C.-A., Loum B., Fall A.-K., Cucherousset J., Ndiaye A.-R. (2017). Kikuchi-Fujimoto disease, a rare cause of lymphadenopathy in Africa. Description of the first case in Senegal and review of the literature. *European Annals of Otorhinolaryngology, Head and Neck Diseases*.

[2] Joean O., Thiele T., Raap M., Schmidt R. E., Stoll M. (2018). Take a second look: it’s Kikuchi’s disease! A case report and review of literature. *Clinics and Practice*.

[3] Perry A. M., Choi S. M. (2018). Kikuchi-Fujimoto disease: a review. *Archives of Pathology & Laboratory Medicine*.

[4] Lahma J., Arkoubi Z., Hejjouji R. (2018). About a rare disease misdiagnosed as malignant lymphoma or tuberculosis: Kikuchi-Fujimoto’s disease. *Pan African Medical Journal*.

[5] Iba Ba J., Nzenze J. R., Missounga L. (2010). Kikuchi-Fujimoto disease in Gabon. Description of first 2 cases in Gabon. *Médecine Tropicale*.

[6] El Mezni F., Mrad K., el Mezni-Benzarti A., Zermani R., Ben Abdeladhim A., Ben Jilani S. (1998). Kikuchi-Fujimoto subacute necrotizing lymphadenitis: two histologic forms observed in the same patient. *Annales de Pathologie*.

[7] Kim T. Y., Ha K.-S., Kim Y., Lee J., Lee K., Lee J. (2014). Characteristics of Kikuchi-Fujimoto disease in children compared with adults. *European Journal of Pediatrics*.

[8] Singh J. M., Shermetaro C. B. (2019). Kikuchi-Fujimoto disease in Michigan: a rare case report and review of the literature. *Clinical Medicine Insights: Ear, Nose and Throat*.

[9] Xu Z., Liu Y., Li H. (2017). Detection of mycobacterial and viral DNA in Kikuchi-Fujimoto disease: an analysis of 153 Chinese pediatric cases. *Science China Life Sciences*.

[10] Tanaka T., Ohmori M., Yasunaga S., Ohshima K., Kikuchi M., Sasazuki T. (1999). DNA typing of HLA class II genes (HLA-DR, -DQ and -DP) in Japanese patients with histiocytic necrotizing lymphadenitis (Kikuchi’s disease). *Tissue Antigens*.

[11] Amir A. R. A., Amr S. S., Sheikh S. S. (2002). Kikuchi-Fujimoto’s disease: report of familial occurrence in two human leucocyte antigen-identical non-twin sisters. *Journal of Internal Medicine*.

[12] Kucukardali Y., Solmazgul E., Kunter E., Oncul O., Yildirim S., Kaplan M. (2007). Kikuchi-Fujimoto disease: analysis of 244 cases. *Clinical Rheumatology*.

[13] Dumas G., Prendki V., Haroche J. (2014). Kikuchi-Fujimoto disease: retrospective study of 91 cases and review of the literature. *Medicine*.

[14] McKenna C., Whitfield T., Patel N., Bonington A. (2017). TB or not to be? Kikuchi-Fujimoto disease: a rare but important differential for TB. *BMJ Case Reports*.

[15] Mahfoudhi M., Gorsane I., Turki S., Abdallah T. B. (2015). Kikuchi-Fujimoto disease mimicking tuberculosis. *International Journal of Clinical Medicine*.

[16] Baenas D., Diehl F., Haye Salinas M., Riva V., Diller A., Lemos P. (2016). Kikuchi-Fujimoto disease and systemic lupus erythematosus. *International Medical Case Reports Journal*.

[17] Goldblatt F., Andrews J., Russell A., Isenberg D. (2007). Association of Kikuchi-Fujimoto’s disease with SLE. *Rheumatology*.

[18] Deaver D., Horna P., Cualing H., Sokol L. (2014). Pathogenesis, diagnosis, and management of Kikuchi-Fujimoto disease. *Cancer Control*.

[19] Dorfman R. F., Berry G. J. (1988). Kikuchi’s histiocytic necrotizing lymphadenitis: an analysis of 108 cases with emphasis on differential diagnosis. *Seminars in Diagnostic Pathology*.

[20] Kido H., Kano O., Hamai A. (2017). Kikuchi-Fujimoto disease (histiocytic necrotizing lymphadenitis) with atypical encephalitis and painful testitis: a case report. *BMC Neurology*.

[21] Jasti D. B., Prasad S. V. N., Naveen T., Vengamma B. (2016). Kikuchi-Fujimoto disease presenting as brainstem encephalitis with secondary blepharospasm. *Journal of Neurosciences in Rural Practice*.

[22] Khishfe B. F., Krass L. M., Nordquist E. K. (2014). Kikuchi disease presenting with aseptic meningitis. *The American Journal of Emergency Medicine*.

[23] Komagamine T., Nagashima T., Kojima M. (2012). Recurrent aseptic meningitis in association with Kikuchi-Fujimoto disease: case report and literature review. *BMC Neurology*.

[24] Lee E. J., Lee H. S., Park J. E., Hwang J. S. (2018). Association Kikuchi disease with Hashimoto thyroiditis: a case report and literature review. *Annals of Pediatric Endocrinology & Metabolism*.

[25] Bousquet E., Tubéry M., Brousset P. (1996). Syndrome de Kikuchi, thyroïdite de Hashimoto et sérologie lupique. À propos d’un cas. *La Revue de Médecine Interne*.

[26] Aqel N. M., Amr S. S., Najjar M. M., Henry K. (1997). Kikuchi’s lymphadenitis developing in a patient with mixed connective tissue disease and Hashimoto’s thyroiditis. *Rheumatology*.

[27] Ahmed H. G. E., Nassar A. S., Ginawi I. (2011). Screening for tuberculosis and its histological pattern in patients with enlarged lymph node. *Pathology Research International*.

[28] Youssef A., Ali R., Ali K., AlShehabi Z. (2017). Kikuchi–Fujimoto disease: a case report of a multi-drug resistant, grueling disease. *Oxford Med Case Reports*.

[29] Barbat B., Jhaj R., Khurram D. (2017). Fatality in Kikuchi-Fujimoto disease: a rare phenomenon. *World Journal of Clinical Cases*.

[30] Song J. Y., Lee J., Park D. W. (2009). Clinical outcome and predictive factors of recurrence among patients with Kikuchi’s disease. *International Journal of Infectious Diseases*.

